# Evaluation des prescriptions antibiotiques au service des urgences de l’Hôpital Militaire d’Instruction Mohammed V (HMIMV)

**DOI:** 10.11604/pamj.2016.25.162.7080

**Published:** 2016-11-16

**Authors:** Anass Elbouti, Mostafa Rafai, Naoufal Chouaib, Said Jidane, Ahmed Belkouch, Hicham Bakkali, Lahcen Belyamani

**Affiliations:** 1 Service des Urgences Médico-chirurgicales, Hôpital Militaire d’Instruction Mohammed V, Faculté de Médecine et de Pharmacie de Rabat, Université Mohammed V, Rabat, Maroc

**Keywords:** Antibiotiques, pertinence et conformité, urgences, Antibiotics, appropriateness and compliance, emergencies

## Abstract

Cette étude à pour objectifs de décrire les pratiques des prescriptions, évaluer leur pertinence et leur conformité aux règles d’utilisations et étudier les facteurs susceptibles de les influencer. Il s’agit d’une étude transversale d’évaluation des prescriptions antibiotiques portant sur 105 patients réalisée au service des urgences médico-chirurgicales de l’H.M.I.Med V de Rabat sur une période d’un mois. Le recueil des données était fait à l’aide d’un questionnaire rapportant les données démographiques et anamnestiques, les antécédents, la notion d’allergie, les données spécifiques de l’examen clinique, les données para cliniques, la prescription détaillée de l’antibiotique. Les données récoltées ont été ensuite évaluées par un médecin référent, chargé d’indiquer les éventuelles erreurs de traitement. Parmi les infections ayant motivé la prescription des antibiotiques, les affections des systèmes respiratoires et urinaires étaient au premier rang, les familles d’antibiotiques les plus couramment employées sont les pénicillines, les quinolones et les céphalosporines. 74 prescriptions soit (70,5%) étaient à la fois pertinentes et conformes contre 9 prescriptions soit (8,6%) justifiées mais non pertinentes et 6 prescriptions soit (5,7%) étaient jugées injustifiées par le médecin référent par absence d’infection. Les évaluations des pratiques médicales sont rarement menées dans les établissements de santé; c’est dans ce cadre que nous avons voulu nous inscrire en produisant cette étude afin d’améliorer la pertinence de nos prescriptions antibiotiques et d’optimiser leur conformité aux différentes recommandations.

## Abstract (en)

The objectives of this study are to describe prescribing practices, to evaluate their appropriateness and their compliance with guidelines and to examine their influencing factors. We conducted a cross-sectional study of antibiotic prescriptions data of 105 patients in the medical and surgical Emergency Department of the Military Hospital of Instruction Mohammed V in Rabat over a period of one month. Data were collected by means of a questionnaire including demographic and anamnestic data, patient’s history, allergies, specific clinical examination data, paraclinical data, detailed antibiotic prescription. Collected data were then evaluated by a referring physician, who was responsible for detecting any treatment error. Among infections requiring the prescription of antibiotics, respiratory and urinary system diseases were at the forefront, the most commonly used antibiotic families were penicillins, quinolones and cephalosporins. 74 prescriptions (70.5%) were both pertinent and compliant versus 9 prescriptions (8.6%) justified but nonpertinent and 6 prescriptions (5.7%) which were considered unjustified by the referring physician due to absence of infection. The evaluation of the existing medical practice is rarely conducted in health facilities; with this in mind, our case study aims to improve appropriate antibiotic prescribing and to optimize its compliance with guidelines.

## Introduction

Les antibiotiques ont été la plus grosse avancée thérapeutique de la médecine dans la seconde moitié du XX^ème^siècle; ils ont permis de sauver d’innombrables vies menacées par des infections autrefois fatales [1]. Dans les services d’urgence l’antibiothérapie est particulièrement fréquente du fait de la prévalence élevée de maladies infectieuses communautaires dans la population consultant ces services, et la prescription des antibiotiques est souvent empirique car les éventuels résultats bactériologiques ne sont pas disponibles au moment de la prescription. Cependant, l’usage non contrôlé des antibiotiques peut avoir deux conséquences: émergence des souches bactériennes résistantes et une morbimortalité importante en rapport avec l’antibiothérapie inadéquate des infections sévères. Une volonté globale de contrôle des prescriptions hospitalières d’antibiotiques est née de ces constats. Les sociétés savantes de pathologies infectieuses publient régulièrement des recommandations consensuelles pour une prescription adaptée à chaque diagnostic [2]. En revanche, les évaluations des pratiques médicales sont rarement menées dans les établissements de santé des pays en développement; c’est dans ce cadre que nous avons voulu nous inscrire en produisant ce travail dont l’objectif était de décrire les pratiques des prescriptions des antibiotiques au service des urgences médico-chirurgicale de l’hôpital militaire de rabat, d’ évaluer leur pertinence et leur conformité aux règles d’utilisations et d’étudier les facteurs susceptibles de les influencer.

## Méthodes

Il s’agissait d’une étude transversale d’évaluation des prescriptions antibiotiques effectuée au sein du service d’accueil des urgences médico-chirurgicales d’un hôpital universitaire (HMIMV Rabat) sur une période de 01 mois. Ont été inclus dans ce travail Tous les patients examinés au service des urgences de l’HMIMV et pour lesquels une antibiothérapie était instaurée, toute la pathologie infectieuse était concernée, antibioprophylaxies comprises, que les patients aient bénéficié ou non d’une antibiothérapie préalable. L’aval du comité d’éthique n’a pas été nécessaire, vu le caractère strictement observationnel de l’étude. L’évaluation a été faite grâce à une fiche d’exploitation préétablie. Chaque questionnaire était remplie par un interne, un résident ou un spécialiste ayant réalisé la prescription ; les données récoltées ont été ensuite évaluées par un médecin référent, chargé d’indiquer les éventuelles erreurs de traitement et de mentionner, en cas de thérapie erronée, le traitement idéal. L’analyse statistique a été faite par le logiciel SPSS pour Windows, version 13 (SPSS, Inc. Chicago, IL, USA). Les résultats ont été exprimés en effectifs (pourcentages) pour les variables qualitatives et en moyenne +/- écarts type et médiane et quartiles pour les variables quantitatives.

## Résultats

Pendant la période d’étude 105 prescriptions ont été recensées, la population se répartissait en 57 hommes (54,3%) pour 48 femmes (45,7%) avec un sexe ratio de (1,18), l’âge moyen était de 40 ans, avec des extrêmes variant de 25 ans et 60 ans, 96 ,2% de nos patients ne présentaient aucune allergie connue aux antibiotiques contre 3,8% en particulier à la pénicilline. 74,3% des prescriptions étaient faites par des médecins internes contre 25,7% des prescriptions par les résidents et les spécialistes du service.

Les diagnostics d’admission étaient dominés par les pathologies respiratoires et urinaires, en fait ; les infections respiratoires basses motivaient 25,7% des prescriptions antibiotiques suivies des pathologies urogénitales 24,8%, des pathologies ORL et respiratoires hautes 18,1%. Alors que les pathologies cutanées, digestives, cérébro-méningées et oculaires se répartissaient 31,4% des prescriptions. Seulement 5 patients ont reçu une antibiothérapie avant leur arrivés aux urgences et 28 patients soit (26,7%) ont été hospitalisé au sein du service des urgences contre 77 traitements en ambulatoire (Tableau 1).

**Tableau 1:** Données concernant l’antibiothérapie = indications de prescription, antibiothérapie antérieure

*Caractéristique*	*Valeur*
Indication de prescription	
Infections ORL et respiratoires hautes	19(18,1)
Infections respiratoires basses	27(25,7)
Infections digestives	10(9,5)
Infections uro-génitales	26(24,8)
Infections ostéo-articulaires	2(1,9)
Infections cutanées	18(17,1)
Infections cérébro-méningées	1(1)
Infections oculaires	2(1,9)
Antibiothérapie antérieure	
Oui	5(4,8)
Non	100(95,2)
Hospitalisation	
Oui	28(26,7)
Non	77(73,3)

**Figure 1:**
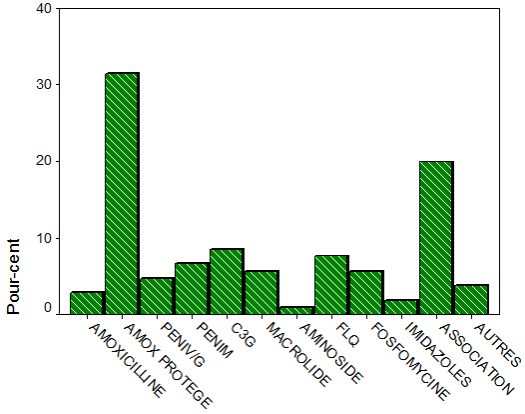
Répartition des prescriptions selon les familles des antibiotiques

Parmi les familles d’antibiotiques prescrites l’amoxicilline protégée occupe 31,4% suivie des céphalosporines 8,6 % et des fluroquinolones7, 6%. Par ailleurs, on note que les associations des différentes familles d’antibiotiques étaient prescrites chez 21 patients soit 20% (Figure 1). La voie orale était utilisée de manière préférentielle avec un total de 71,4% contre 26,7% de prescriptions en intraveineux et 1,9% des prescriptions par voie locale.

**Figure 2:**
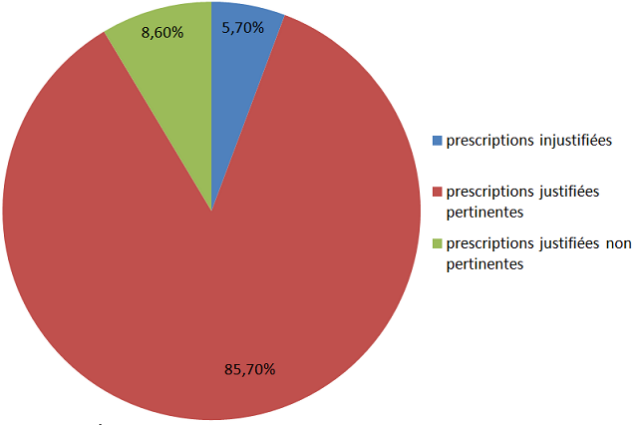
Évaluation de la pertinence des prescriptions

En ce qui concerne la pertinence des prescriptions; 6 prescriptions soit (5 ,7%) étaient jugées injustifiées par le médecin référent par absence d’infection, parmi les 99 prescriptions restantes ,90 soit (85,7%) étaient à la fois justifiées et pertinentes contre 9 prescriptions (8 ,6%) justifiées mais non pertinentes (Figure 2).

**Figure 3:**
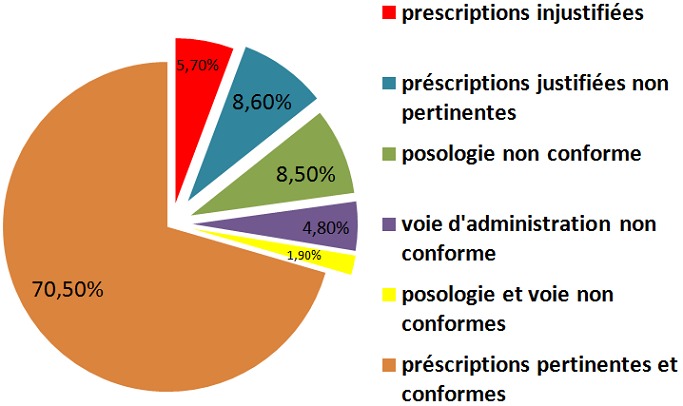
Évaluation de la pertinence et la conformité des prescriptions

Sur 90 prescriptions jugées justifiées et pertinentes ,16 prescriptions (15,2%) ont été non conformes, les erreurs de non-conformité se partageaient entre la posologie et la voie d’administration; la posologie été jugée non conforme dans 9 prescriptions, alors que la voie d’administration été non conforme dans 5 prescriptions (4,8%). Seulement 2 prescriptions avaient à la fois une posologie et une voie d’administration non conformes et 74 prescriptions soit 70,5% ont été jugées à la fois conformes et pertinentes (Figure 3).

En analyse univariée, La pertinence et la conformité de la prescription des antibiotiques n’étaient pas liées de manière significative aux caractéristiques sociodémographiques des patients. Par ailleurs, On a conclu à une différence statistiquement significative dans la mesure où le statut du prescripteur (interne versus résident /spécialiste) est un facteur influençant la conformité et la pertinence des prescriptions, cela s’explique en partie par le fait que les internes se référent toujours, en cas de doute, a un senior (Tableau 2). L’indication de prescription de l’antibiotique était également un facteur influençant la conformité, en fait ; les erreurs de prescription concernent surtout les infections respiratoires basses, urogénitales et digestives (Tableau 3). On a conclu aussi à une différence statistiquement significative en termes de choix de la famille de l’antibiotique prescrit et la conformité ; deux causes majeures de non pertinence des antibiothérapies ont été identifiées : la prescription d’une molécule inadaptée et la mise en route d’associations non recommandées (Tableau 4).

**Tableau 2:** Relation entre le protocole antibiotique prescrit (conforme ou non) et les données générales liées à l’antibiothérapie

*Caractéristiques*	*Protocole conforme d’antibiotique*		*p*
	*Oui (N= 74)*	*Non (N= 31)*	
**Antibiothérapie antérieure**			0,138
Oui	5(100)	0(0)	
Non	69(69)	31(31)	
**Voie d’administration**			0,299
Intraveineuse	55(73,3)	20(26,7)	
Orale	17(60,7)	11(31,3)	
Locale	2(100)	0(0)	
**Prescripteur**			*0,01*
Médecin interne	60(76,9)	18(23,1)	
Médecin résident	14(51,9)	13(49,1)	
Spécialiste	13(49,1)		

Les valeurs sont exprimées en moyenne+/-écart-type et effectif (pourcentage); Le seuil de significativité statistique choisi (p=0,05). Le test Khi-deux à été utilisé pour les variables qualitatives.

**Tableau 3:** Relation entre le protocole antibiotique prescrit (conforme ou non) et indication de prescription de l’antibiothérapie

*Caractéristiques*	*Protocole conforme d’antibiotique*		*p*
	*Oui (N= 74)*	*Non (N= 31)*	
**Indication de prescription**			*<0,01*
Infections ORL et respiratoires hautes	16(84,2)	3(15,8)	
Infections respiratoires basses	18(66,7)	9(33,3)	
Infections digestives	1(10)	9(90)	
Infections uro-génitales	20(76,9)	6(23,1)	
Infections ostéo-articulaires	2(100)	0(0)	
Infections cutanées	14(77,8)	4(22,2)	
Infections cérébro-méningées	1(100)	0(0)	
Infections oculaires	2(100)	0(0)	

Les valeurs sont exprimées en moyenne+/-écart-type et effectif (pourcentage) ; Le seuil de significativité statistique choisi (p=0,05). Le test Khi-deux qui à été utilisé.

**Tableau 4:** Relation entre le protocole antibiotique prescrit (conforme ou non) et l’antibiotique prescrit par famille

*Caractéristiques*	*Protocole conforme d’antibiotique*		*p*
	*Oui (N= 74)*	*Non (N= 31)*	
Antibiotiques prescrits par famille			*0,04*
Amoxicilline	2(66,7)	1(33,3)	
Amoxicilline protégée	24(72,7)	9(27,3)	
Péni V/G	4(80)	1(20)	
Péni M	7(100)	0(0)	
C3G	8(88,9)	1(11,1)	
Macrolide	2(33,3)	4(66,7)	
Aminoside	1(100)	0(0)	
Fluoroquinolone	7(87,5)	1(12,5)	
Fosfomycine	4(66,7)	2(33,3)	
Imidazolés	0(0)	2(100)	
Autres	4(100)	0(100)	
Associations	11(52,4)	10(47,6)	

Les valeurs sont exprimées en moyenne+/-écart-type et effectif (pourcentage) ; Le seuil de significativité statistique choisi (p=0,05). Le test Khi-deux qui à été utilisé.

## Discussion

Les évaluations des pratiques professionnelles sont devenues obligatoires. De nombreux travaux ont démontré l’importance de ces évaluations en termes d’amélioration de la pertinence et de la conformité des prescriptions antibiotiques et en termes de réduction des dépenses [3]. C’est dans cette perspective que nous avons voulu nous inscrire en produisant cette étude, afin d’optimiser au mieux nos prescriptions. En fait ; Dans la présente étude, 74 prescriptions soit (70,5%) étaient à la fois pertinentes et conformes contre 9 prescriptions soit (8,6%) justifiées mais non pertinentes et 6 prescriptions soit (5,7%) étaient jugées injustifiées par le médecin référent par absence d’infection. Concernant le taux de pertinence Ce chiffre est élevé comparativement à une étude réalisée dans le service des urgences du centre hospitalier universitaire Ibn Sina de Rabat qui a montré que seulement 45,5% des prescriptions antibiotiques étaient appropriées [4], dans les autres études de la littérature le taux de pertinence est compris entre 30 et 80% selon les études [5–8]. Il est probable que remplir le questionnaire ait eu un effet en soi permettant une meilleure pertinence des prescriptions (effet Hawthorne) [9].

Après avoir évalué la pertinence des antibiotiques, le médecin référent s’est attaché à évaluer la posologie et la voie d’administration des prescriptions jugées justifiées et pertinentes. Une posologie non conforme été constatée dans 9 prescriptions soit (8,5%), une voie d’administration non conforme été retrouvée dans 5 prescriptions soit (4,8%) et elle s’agissait de la voie intraveineuse qui été choisie à tort au détriment de la voie orale, notamment dans les infections respiratoires hautes. Il est à noter toutefois, que la durée des traitements antibiotiques et le devenir des patients n’ont pas été relevés au cours de notre étude.

La pertinence des prescriptions antibiotiques était liée de manière significative à l’indication de prescription, le choix de l’antibiotique et de l’association (p=0,04), et surtout le statut du prescripteur (p=0,01). Pour ce qui est de l’influence du prescripteur, le taux de non-conformité était de 23% dans les prescriptions faites par les internes tandis que la non-conformité était proche de 50% dans les prescriptions des résidents et spécialistes du service, et ce en opposition à deux études qui associaient le fait d’être interne à un taux de pertinence des prescriptions moins bon [7, 8], et concorde avec l’étude realisée par Asseray.N et al [10], alors qu’il n’existe pas de lien entre le taux de pertinence et le statut du prescripteur dans l’étude de *Gennai S et al* [5].

L’influence de la qualité du prescripteur sur la pertinence et la conformité des prescriptions, retrouvée dans notre enquête, peut être expliquée en partie par le fait que, les internes disposent d’un droit de prescription limité dans le SAU de l’HMIMV, et se référent toujours, en cas de doute, a un senior. Mais le nombre réduit de notre série constitue une limite statistique pour conclure à des résultats interprétables.

## Conclusion

La prescription d’une antibiothérapie aux urgences repose souvent sur un raisonnement probabiliste qui doit être très rigoureux du fait des implications pronostiques potentielles et immédiates. Il importe donc que les médecins urgentistes reconnaissent précocement ces situations et y répondent de façon adaptée en se basant sur les protocoles d’antibiothérapies recommandées par les conférences de consensus.

### Etat des connaissances actuelles sur le sujet

- Dans les services d’urgence l’antibiothérapie est particulièrement fréquente du fait de la prévalence élevée de maladies infectieuses communautaires dans la population consultant ces services;
- La prescription des antibiotiques est souvent empirique car les éventuels résultats bactériologiques ne sont pas disponibles au moment de la prescription;
- Les sociétés savantes de pathologies infectieuses publient régulièrement des recommandations consensuelles pour une prescription adaptée à chaque diagnostic.

### Contribution de notre étude à la connaissance

- Décrire les pratiques des prescriptions des antibiotiques dans un service des urgences;
- Evaluer la pertinence et la conformité des prescriptions aux règles d’utilisations;
- Etudier les facteurs susceptibles de les influencer.
